# Intrathecal Co-administration of Morphine Facilitated the Anti-nociceptive of Bupivacaine in a Rat Model of Acute Postoperative Pain

**DOI:** 10.7759/cureus.28385

**Published:** 2022-08-25

**Authors:** Tamoghna Ghosh, Subrata Basu Ray

**Affiliations:** 1 Department of Medicine, All India Institute of Medical Sciences, New Delhi, New Delhi, IND; 2 Department of Anatomy, All India Institute of Medical Sciences, New Delhi, New Delhi, IND

**Keywords:** local anaesthetic, rat model, post incisional pain, mechanical allodynia, analgesia

## Abstract

Introduction: Bupivacaine is one of the commonly used agents for spinal anaesthesia. Moreover, co-administration with morphine can likely increase its anti-nociceptive effect bringing about a reduction in the required dose of bupivacaine. Though this has been observed clinically, preclinical studies on the efficacy of this drug combination are lacking.

Methods: Sprague Dawley rats, previously implanted with intrathecal catheters, were administered either bupivacaine (30 mcg) or morphine (30 mcg) or both bupivacaine and morphine (15 mcg each). These doses were determined following prior evaluation of different doses of bupivacaine (3, 10 and 30 mcg). Rats were subjected to hind paw incision under isoflurane anaesthesia, 15 min after drug administration. Anti-nociception was evaluated by estimating mechanical allodynia in a fixed peri-incisional area using von Frey filaments. This was done 4 h after the incision.

Results: Both bupivacaine and morphine attenuated allodynia though morphine was more effective. Co-administration of both drugs at half the doses increased the antinociceptive effect of bupivacaine to the 30 mcg dose level.

Conclusion: The underlying reason for this enhanced anti-nociception could be the different neural mechanisms responsible for anti-nociception. Local anaesthetics inhibit the generation of action potentials by blocking sodium channels whereas opioids like morphine act through G-protein coupled mu opioid receptor-linked closure of calcium channels in presynaptic terminals. In conclusion, the addition of morphine can facilitate bupivacaine’s anti-nociceptive effect following intrathecal administration. This information could have clinical relevance in the treatment of postoperative pain.

## Introduction

Bupivacaine is a potent, long-acting local anaesthetic (LA) agent, commonly used for spinal anaesthesia [1,2]. It blocks voltage-gated sodium channels in the nerve roots and dorsal root ganglia following neuraxial administration. Morphine is a prototypical opioid drug, which closes voltage-gated calcium channels by binding to G protein-coupled mu-opioid receptors. These receptors are predominantly expressed in Rexed’s laminae I-II of the spinal cord [3]. Considering their different mechanisms of action, it was hypothesized that co-administration of bupivacaine and morphine could increase the overall analgesic effect. It may also lower the requirement of bupivacaine leading to a decrease in the incidence of side effects like arrhythmias and cardiac arrest. In a clinical study on 90 subjects, patients who had received both bupivacaine and morphine showed lower VAS scores after lower limb orthopaedic surgery [4]. Also, consumption of bupivacaine was less during the postoperative period.

To the best of my knowledge, there are no existing preclinical studies on the efficacy of this drug combination on rodent postoperative pain models. Although, intrathecal administration of bupivacaine (200 mcg/animal) or morphine (5 and 30 mcg/animal) alone has been shown to reduce incision-induced allodynia [5]. A drawback was the high dose of bupivacaine used in this earlier study, which could lead to motor weakness and even paralysis. In turn, this could interfere with the behavioural testing for pain which depends on the withdrawal of the affected paw from the painful stimulus. Consequently, in this work, lower doses of bupivacaine (3, 10 or 30 mcg/animal) were tested and the 30-mcg dose was selected for the final study. The dose of morphine was the same (30 mcg) as that used in a previous study in our laboratory [6].

The hind paw incision rodent model for postoperative pain was first described in 1996 [7]. Subsequently, this model has been used extensively for evaluating basic mechanisms of postoperative pain as well as the efficacy of various analgesic drugs. The animals used in the present study were previously implanted with intrathecal catheters following earlier described procedure [8]. After pre-emptive drug administration (bupivacaine or morphine or both) via the catheter, rats were subjected to paw incision. Postoperative pain was estimated at 4 h after surgery by determination of mechanical allodynia (hypersensitivity to non-noxious mechanical stimuli) using graded von Frey filaments [9]. This time point was selected based upon adequate recovery from surgical procedure and corresponded with a high degree of pain in the animals.

## Materials and methods

Experimental animals

Adult male Sprague-Dawley rats, weighing 275-325 g were used in the study (n=30). Permission was obtained from the Institutional animal ethics committee prior to the work. Animals were provided food and water ad libitum. After surgery, the bedding material in cages was changed to a clean cellulose-based material known as Alpha-dri (Shepherd speciality papers, USA). This was done to protect the incised paw. 

Intrathecal placement of a catheter

**Figure 1:**
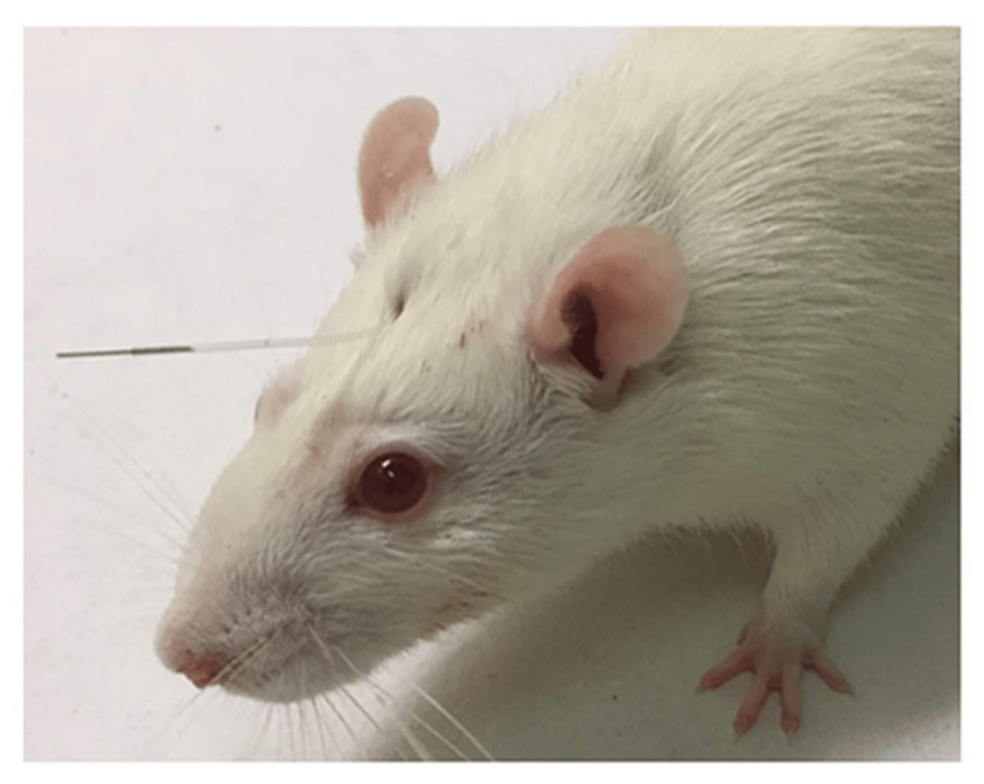
A rat with a surgically implanted intrathecal catheter.

Sterile, polyethylene catheters, individually packed, were purchased from ReCath Company, USA. The catheters were implanted through the cisternal membrane in the suboccipital region under isoflurane anaesthesia (Vet-Tech instruments, UK) [8]. It is depicted in Figure 1. The in-dwelling end was gradually pushed into the intrathecal space so that 8.5 cm of the catheter was introduced. In the end, the distal end was lodged just above the lumbar enlargement of the spinal cord. The out-dwelling end of the catheter was exteriorized through the back of the head. It was plugged with a stainless wire to prevent CSF leakage. On day 3 after implantation, 15 µL of 2% lignocaine was administered through the catheter to check its location. There was temporary paralysis of the hind paws for about 5-10 mins. Experimental procedures were started one week after implantation.

Drug administration

Morphine sulphate I.P. injection (15 mg/mL) was purchased from the Government Agency (Verve health care, New Delhi). Bupivacaine hydrochloride I.P. (5 mg/mL) was sourced from a local pharmacy (Troikaa Pharmaceuticals Ltd., Gujarat). These were diluted in physiological saline. Both the drugs were mixed in saline before co-administration. The total volume of drug injected was 10 µL in each animal. The experimental work was done in two stages. In the initial part, different doses of bupivacaine (3, 10 and 30 µg) were administered by a Hamilton syringe (n=9 rats; three/group) under light physical restraint. 15 min later, these animals were subjected to paw incision (described below). 4 h after incision, these animals were evaluated for allodynia. Based on the results, the 30-µg dose was selected for further study.

In the second part, rats were divided into four equal groups (six rats/group) and treated with saline (control group), bupivacaine (30 µg), morphine (30 µg) and bupivacaine + morphine (15 µg of each) by the intrathecal route. Again, after 15 min later, the animals were subjected to paw incision and allodynia was determined in the peri-incisional area using the up-down method at 4 h.

Thus, we involved a total of 30 rats (six in saline control, six in the 30 µg morphine group, and six (for the first part of the experiment, only three were used, for the second part, three more were added) in 30 µg bupivacaine group, three in 10 µg bupivacaine group, three in 3 µg bupivacaine group, and six in bupivacaine + morphine group) in our study.

The procedure of paw incision

**Figure 2:**
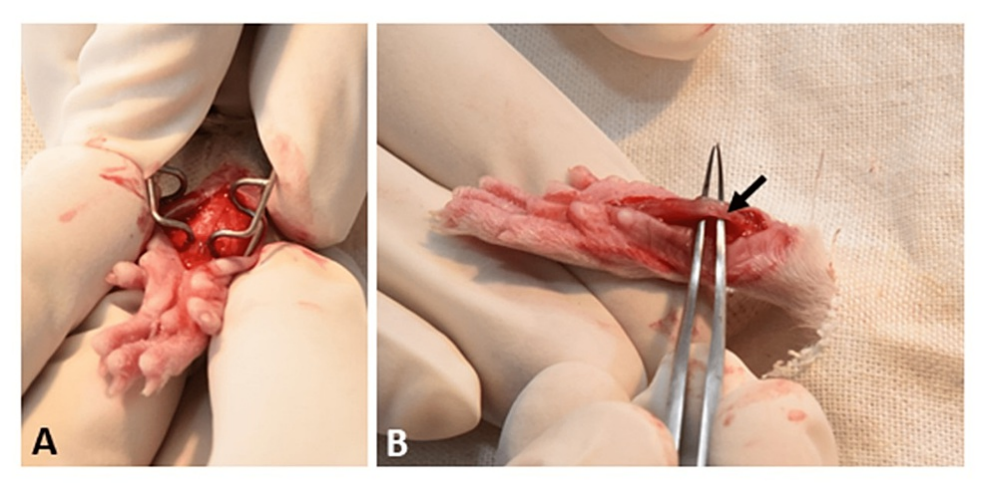
Procedure of hind paw incision. (A) The skin edges were retracted for exposing the flexor digitorum brevis. (B) The muscle was elevated and incised.

Following inhalation anaesthesia, rats were placed in a prone position and the plantar surface of the right paw taken out through a drape as depicted in Figures 2A, 2B. It was swabbed with Povidone-iodine solution and isopropyl alcohol. Then, starting 0.5 cm from the proximal end of the heel, a 1-cm long skin incision was made using a no. 11 scalpel blade [7]. The skin was retracted and the underlying muscle was lifted and incised longitudinally for 0.5 cm. The tip of a forceps was passed through the cut in the muscle and the blades separated slightly. After haemostasis, the skin edges were apposed by two mattress sutures with the knots placed on the lateral side. Neosporin ointment was applied to the incision site. Rats were transferred to a warm recovery chamber till they regained consciousness. Finally, they were transferred to their home cages, where they were kept singly.

Behavioural assessment of pain

Rats were placed on an elevated wire mesh platform (8x8 mm gaps) and covered with Perspex enclosures (16x16x16cm). After 30 min of acclimatization, calibrated nylon filaments of different sizes (3.61, 3.84, 4.08, 4.31, 4.56, 4.74, 4.93, 5.18) were used for assessing allodynia according to the up-down method [9]. These filaments exert pressure between 0.4 and 15 g when pressed on the paw. Normally, rats do not respond to these filaments. The first filament applied was of size 4.31. The tip was applied to the peri-incisional area (medial side, towards the proximal end of the heel), and pressed till there was buckling of the filament. A positive response was a reflex withdrawal of the paw. Then, four filaments were applied successively after the first one that produced a response. A 50% withdrawal threshold (g) was calculated using an algorithm. Higher values of 50% withdrawal threshold (g) indicate a lesser degree of nociception.

Statistical analysis

The data were analyzed by one-way analysis of variance followed by Tukey’s multiple comparison test (GraphPad Prism, version 8). Values were expressed as mean ± sem. P<0.05 was considered to be significant. Values of the dose-response study were not analyzed as the number of animals was not sufficient.

## Results

Standardization of dose

**Figure 3:**
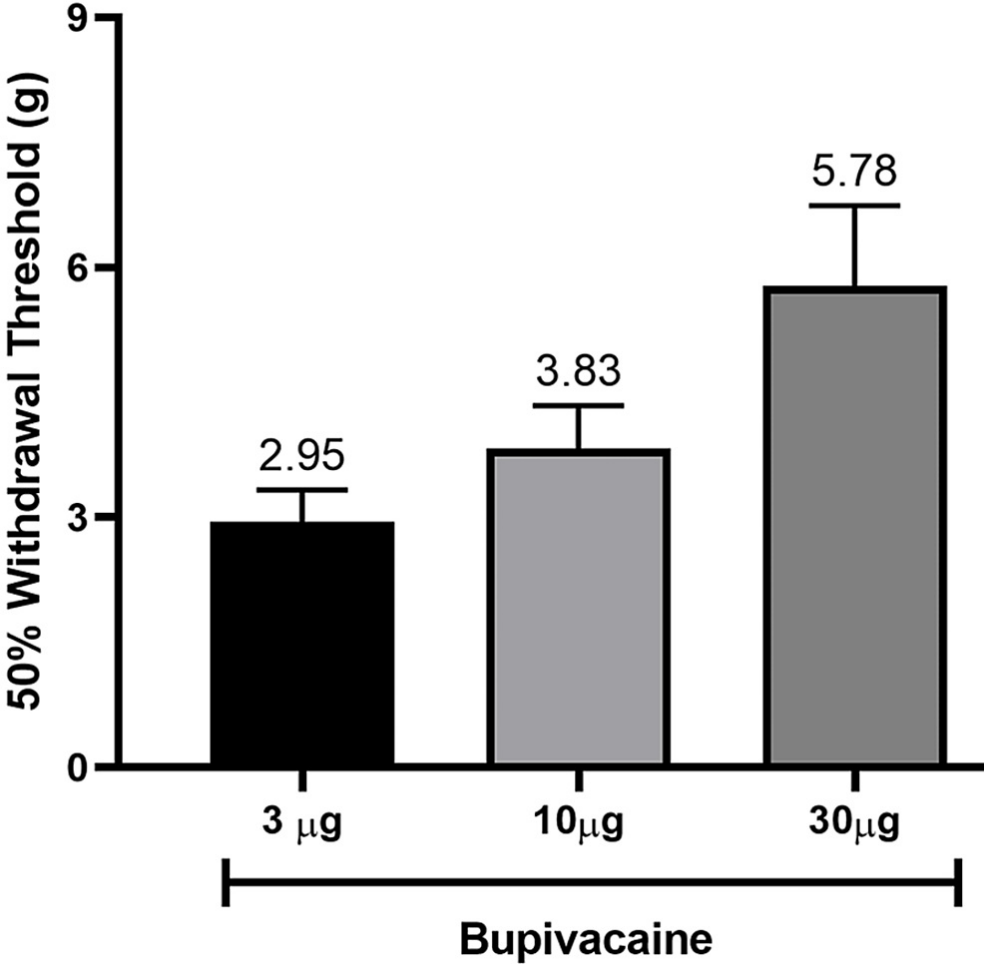
Bar diagram showing the dose-response data for different doses of bupivacaine, statistical analysis was not performed as the number of animals was not sufficient (n=3/dose).

Following intraspinal bupivacaine (3, 10 and 30 µg doses), there was a progressive increase in the anti-nociceptive response as evident from higher values of 50% withdrawal threshold as illustrated in Figure 3. The threshold value of the 30-µg dose was 5.8 ± 0.9 g, which was selected for further study.

Comparative assessment of the anti-nociceptive effect

**Figure 4:**
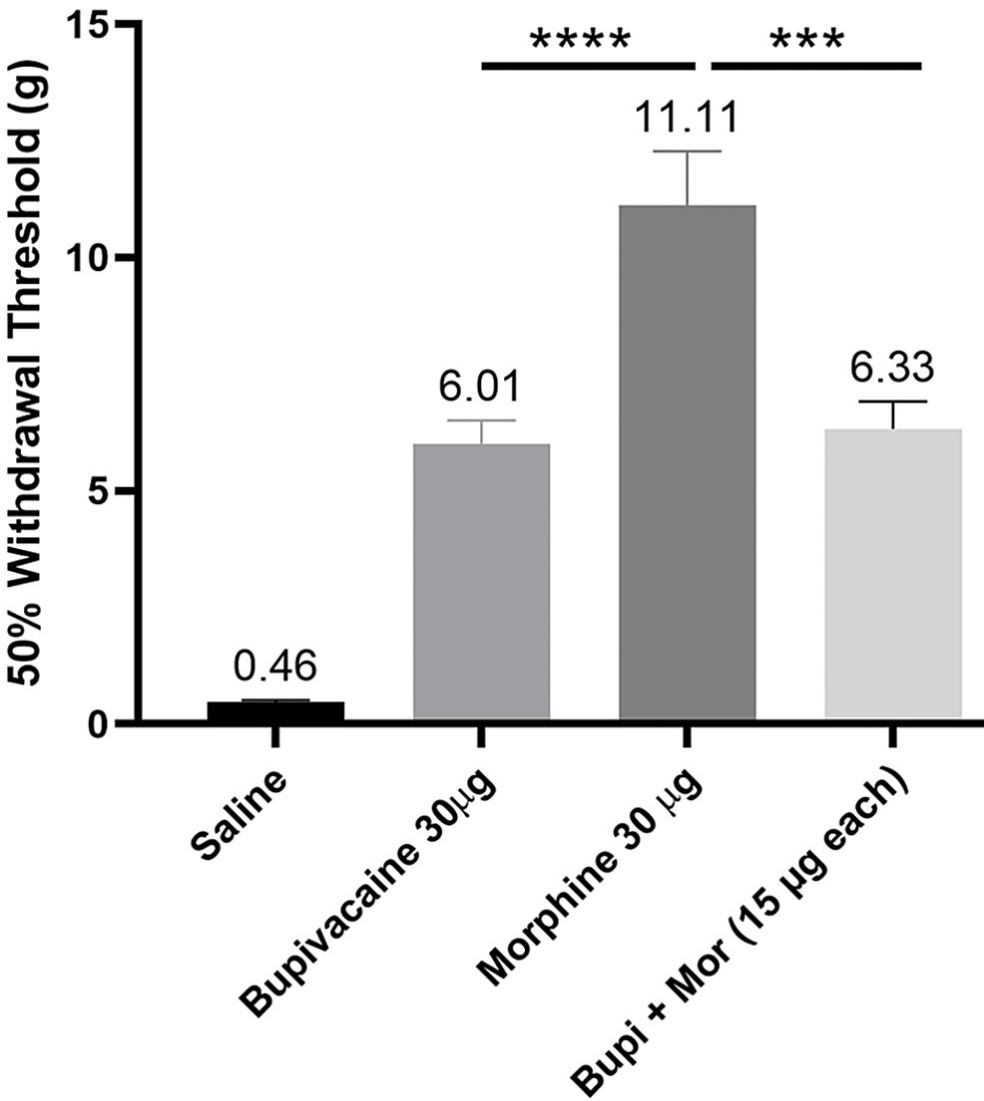
Saline showed minimum value for the 50% withdrawal threshold (g) indicating high extent of allodynia; the up-down test can detect values between 0.4-15 g; all the drug-treated groups showed higher analgesia compared to saline; bupivacaine-morphine combination (15 µg) showed similar anti-nociceptive effect to that of bupivacaine alone (30 µg); interestingly, morphine treatment produced higher anti-nociception than either bupivacaine or bupivacaine + morphine treatment.

Control group showed almost the maximum possible allodynia (threshold value: 0.46±0.05 g; 95% CI - 0.35 to 0.58) as illustrated in Figure 4. All the other groups showed significantly higher values of withdrawal threshold, indicating anti-nociception. Bupivacaine alone produced a moderate effect (6±0.6 g; 95% CI - 4.8 to 7.2) which was significantly less than morphine (11.1±1 g; 95% CI - 8.1 to 14.1). On combining bupivacaine with morphine (15 µg of each), the analgesic effect (6.3±0.6 g; 95% CI - 4.9 to 7.8) was similar to 30 µg of bupivacaine. These values were not significantly different. Thus, morphine facilitated the analgesic effect of bupivacaine.

## Discussion

Among the spinal analgesics used in clinical practice, bupivacaine is perhaps the most widely used [10]. There can be side effects like numbness, paresthesia, weakness and dysfunction of the bladder and bowel. It can also produce cardiovascular and neurological toxicity. In the present study, an attempt was made to reduce the dose of LA by adding morphine in a 1:1 ratio (15 µg each of bupivacaine and morphine). The overall anti-nociceptive effect of the combination therapy was equivalent to a 30-µg dose of bupivacaine. No untoward adverse effect was observed in the animals. Mixtures of these two drugs have been previously reported to be stable [11].

Mechanical allodynia is an important feature of postoperative pain and is robustly demonstrated in the hind paw incision model. This was attenuated by both bupivacaine and morphine treatment. Moreover, co-administration of morphine facilitated the anti-nociceptive of bupivacaine and this is likely the first report on this subject. Previously, a synergistic effect was noted between bupivacaine and morphine following long-term administration in cancer patients [12]. Again, other clinical studies on this drug combination also observed a facilitated analgesic effect [13-15]. An earlier study in rodents also noted a decrease in allodynia following either bupivacaine or morphine administration [5]. This combination of drugs was also found to be effective in relieving neuropathic pain [16].

Also, one of the major patient concerns undergoing inguinal hernia repair is postoperative pain and the need to return to work and daily activity as soon as possible [17]. The goal of postoperative pain management is to reduce or eliminate pain and discomfort with the least side effects and minimal cost. Various drugs are used for postoperative pain management [18]. Parecoxib and acetaminophen are non-opioid analgesics with a well-documented efficacy after different surgical procedures [19]. The use of non-opioid analgesics can reduce opioid-induced side effects like respiratory depression.

## Conclusions

In conclusion, the current study reports that bupivacaine and morphine combination administered through the intrathecal route can be beneficial in the treatment of postoperative pain and also permits a reduction in the total dose of bupivacaine. This information might have clinical relevance. These anesthetics act by different mechanisms with Bupivacaine inhibiting the generation of action potentials by blocking sodium channels whereas opioids, like morphine, act through G-protein coupled mu-opioid receptors and close calcium channels in presynaptic terminals. So, a reduced dose of each drug in the combination decreases their side effects in a dose-dependent manner. Thus, the cardiotoxic effect of bupivacaine and the respiratory depression effect of morphine can be reduced by this method, without compromising the analgesic efficacy.
